# New Perspectives on Myeloid-Derived Suppressor Cells and Their Emerging Role in Haematology

**DOI:** 10.3390/jcm11185326

**Published:** 2022-09-10

**Authors:** Nikoleta Bizymi, Andreas M. Matthaiou, Angelos Matheakakis, Ioanna Voulgari, Nikoletta Aresti, Konstantina Zavitsanou, Anastasios Karasachinidis, Irene Mavroudi, Charalampos Pontikoglou, Helen A. Papadaki

**Affiliations:** 1 Department of Haematology, University Hospital of Heraklion, 71500 Heraklion, Crete, Greece; 2 Haemopoiesis Research Laboratory, School of Medicine, University of Crete, 71003 Heraklion, Crete, Greece; 3 Laboratory of Molecular and Cellular Pneumonology, School of Medicine, University of Crete, 71003 Heraklion, Crete, Greece; 4 Respiratory Physiology Laboratory, Medical School, University of Cyprus, 2029 Nicosia, Cyprus

**Keywords:** myeloid-derived suppressor cell (MDSC), haematology, immunology, cancer, infection, autoimmunity, inflammation, immunotherapy, immune dysregulation, biomarker

## Abstract

Myeloid-derived suppressor cells (MDSCs) are immature cells of myeloid origin that have gained researchers’ attention, as they constitute promising biomarkers and targets for novel therapeutic strategies (i.e., blockage of development, differentiation, depletion, and deactivation) in several conditions, including neoplastic, autoimmune, infective, and inflammatory diseases, as well as pregnancy, obesity, and graft rejection. They are characterised in humans by the typical immunophenotype of CD11b^+^CD33^+^HLA-DR^–/low^ and immune-modulating properties leading to decreased T-cell proliferation, induction of T-regulatory cells (T-regs), hindering of natural killer (NK) cell functionality, and macrophage M2-polarisation. The research in the field is challenging, as there are still difficulties in defining cell-surface markers and gating strategies that uniquely identify the different populations of MDSCs, and the currently available functional assays are highly demanding. There is evidence that MDSCs display altered frequency and/or functionality and could be targeted in immune-mediated and malignant haematologic diseases, although there is a large variability of techniques and results between different laboratories. This review presents the current literature concerning MDSCs in a clinical point of view in an attempt to trigger future investigation by serving as a guide to the clinical haematologist in order to apply them in the context of precision medicine as well as the researcher in the field of experimental haematology.

## 1. Introduction

Myeloid-derived suppressor cells (MDSCs) are a heterogeneous population of cells, identified by the immunophenotype CD11b^+^CD33^+^HLA-DR^–/low^ in humans and divided into CD14^+^ (monocytic, M-MDSCs), CD15^+^ (granulocytic or polymorphonuclear, G- or PMN-MDSCs), and CD14^-^/CD15^-^ (early stage, eMDSCs) MDSCs. In mice, they are divided into Gr1^+^CD11b^+^Ly6G^+^Ly6C^low^ (PMN-MDSCs) and Gr1^+^CD11b^+^Ly6G^-^Ly6C^hi^ (M-MDSCs) [1]. They are immature myeloid cells with an activation profile that deviates from the standard pathway of differentiation, mostly during emergency myelopoiesis in chronic inflammation, autoimmune diseases, and tumour progression, and possess immune-modulating properties [2]. They can also play an ambiguous role in the myeloid microenvironment. While their expansion may promote carcinogenesis through angiogenesis, metastasis, and immune-suppression, as well as chronic inflammation in infections, aging, and neurodegenerative diseases, their existence is also essential for the foetal–maternal immune-tolerance in pregnancy, the graft tolerance in allo-transplantation, and the avoidance of autoimmunity [3,4].

MDSCs have gained the attention of researchers and clinicians during recent years; therefore, there is an abundance of ongoing studies concerning their implication in almost every trending research field: in the tumour microenvironment (TME) communicating with various cell types [5]; in cancer cachexia, where their expansion may be a contributing factor [6]; in tumour progression due to their secreting exosomes and micro-RNAs [7]; in autophagy, which regulates their immune-suppression properties [8]; in immunosenescence and aging where their increase is associated with low-grade chronic inflammation and impairment of the clearance of senescent cells [9,10,11,12]; in metabolomics and energy metabolism showing a unique metabolic profile associated with their functionality and maturation [13,14]; in gut microbiome, which may affect the expansion of PMN-MDSCs [15]; in foetal–maternal tolerance and breastfeeding by accumulating in the maternal and foetal organisms and the breast milk [16,17,18]; in infant infections, as they surprisingly present antimicrobial properties and a particular protective role in newborns [19,20]; in sepsis, where they can serve as important biomarkers and therapeutic targets [21]; even in severe COVID-19, where MDSC populations seem to be upregulated and may predict worse outcomes [22]. Additionally, there are preliminary data that MDSCs may affect the sex dimorphism and differences between genders in the survival rates and response to treatments in various conditions, as they may present different levels in male versus female subjects [23].

The research in the field is challenging, as there are still difficulties in defining cell-surface markers and gating strategies that uniquely identify the different populations of MDSCs [24], whereas the currently available functional assays are highly demanding [25]. New methods, i.e., proteomic and transcriptomic analysis [26], as well as in situ imaging with 3D microscopy and 2D immunofluorescence [27], can further facilitate their study. There is evidence that MDSCs display altered frequency and/or functionality and could be targeted in immune-mediated and malignant haematologic diseases, although there is large variability in techniques and results between different laboratories [28]. This review presents the current literature in a clinical point of view in an attempt to trigger future investigation by serving as a guide to the clinical haematologist as well as the researcher in the field of haematology.

## 2. When to Call a Cell “MDSC”?

The term MDSCs was proposed for the first time in 2007 to substitute the earlier-used term “myeloid suppressor cells” in order to better describe the heterogeneity, origin, biological role, and functionality of these cells [29,30]. According to the last recommendations, for the characterisation of a cell population as MDSCs, the typical immunophenotype, functional characteristics, and/or expression of certain molecules and biochemical markers are needed. More specifically, Bronte et al. suggested that cells demonstrating the typical immunophenotype and molecular and biochemical characteristics, but lacking the immunosuppressive functionality, should rather be characterised as MDSC-like cells (MDSC-LC) [24]. The reason for this recommendation is that MDSCs are found in different stages of differentiation and activation in patients with cancer and inflammation, and probably acquire their suppressive character in later stages of disease [24]. However, the term MDSC-LC is rarely used in the literature, whereas occasionally it is also used to characterise mature cells that acquire immunosuppressive properties typically found in MDSCs. Umemura et al., for example, characterised as MDSC-LC the murine Gr-1^hi^ IL-4Rα^hi^ cells that presented no suppressive properties against CD8^+^ T-cells [13]. On the other hand, Li et al. used the term G-MDSC-LC in order to refer to a population of PMN-MDSCs that can be contaminated with mature neutrophils with suppressive properties [15].

## 3. Where do MDSCs Originate from?

**Figure 1:**
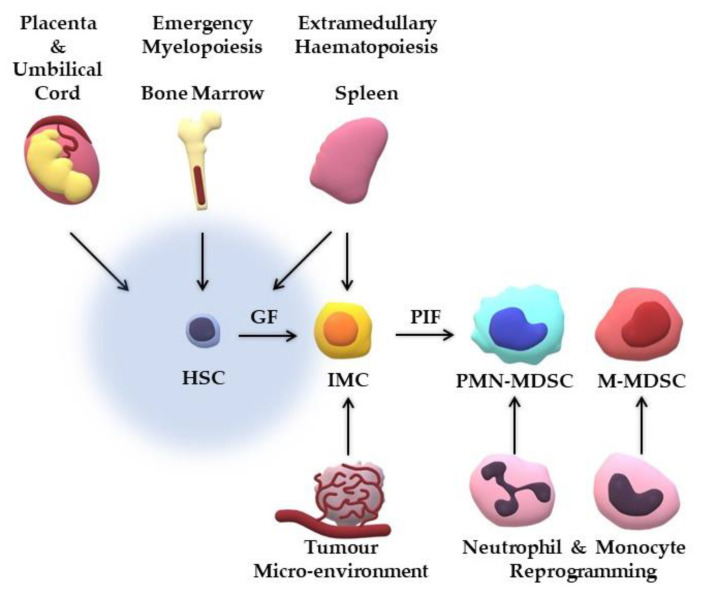
Theories of the origin of MDSCs. The classical theory of the generation of MDSCs is the “two-signal model”. The HSC under the effect of GFs becomes IMC, which under the effect of PIFs turns into an activated with suppressive character cell, i.e., MDSC. Besides the bone marrow, there are also other sites of generation of these cells such as at the site of the tumour, in the spleen, and in the placenta and umbilical cord. Another theory is the reprogramming under specific triggers of mature neutrophils and monocytes to PMN-MDSCs and M-MDSCs, respectively. Abbreviations: HSC—haematopoietic stem cell; IMC—immature myeloid cell; GF—growth factors; PIF—pro-inflammatory factors; MDSC—myeloid-derived suppressor cell; PMN-MDSC—polymorphonuclear-MDSC; M-MDSC—monocytic-MDSC.

The theories for the origin of MDSCs are still diverse. The questions to be answered are numerous and concern the site of their generation, the factors needed for their development, their progenitor forms, etc. [31]. In Figure 1, we summarise the main theories of the origin of MDSCs, and examples of studies investigating their generation are provided in Table 1. However, it is possible that more than one theory is correct and that MDSCs are generated in different ways according to their microenvironment, either normally and with beneficial properties, or abnormally and with deleterious effects on homeostasis.

There is no doubt that key players for their generation are the colony stimulating factors (CSFs), as indicated by the ex vivo expansion of MDSCs from progenitor cells in cultures containing them [32,33,34,35]. Especially, granulocyte-CSF (G-CSF) is thought to drive haematopoiesis to the myeloid lineage in the bone marrow (BM) [36]. The classical theory, called the “two-signal model” and firstly introduced by Gabrilovich et al., suggests the development of the MDSC from the haematopoietic stem cell (HSC) during emergency myelopoiesis in a two-step process. Initially, the HSC, under the exposure to growth factors, mainly CSFs, transforms to an immature myeloid cell (IMC). Finally, under the exposure to pro-inflammatory factors, including signal transducers and activators of transcription (STATs), especially STAT3, S100 calcium-binding protein A8/A9 (S100A8/A9), nuclear factor-kB (NF-kB), retinoic-acid-related orphan receptor C1 (RORC1/RORγ) [37,38], and CCAAT/enhancer-binding protein b (C/EBPb), the IMC pauses its differentiation, becomes activated, and acquires its MDSC character [31,36,37]. When the cell is taken away from the malignant microenvironment, it may be further developed into a more mature form.

Besides the BM, other sites and organs are also responsible for the generation of MDSCs. Even on the site of the tumour or on newly formed pre-metastatic niches, progenitor cells that originated from the BM may transform to IMCs and then activated MDSCs. Spleen, as the major representative of extramedullary haematopoiesis under signals such as lipopolysaccharide (LPS) or interferon-γ (IFN-γ), is one of these sites. MDSCs in the spleen may be either the activated forms of IMCs that initiated from the BM and reached the spleen through the periphery, or *de novo* (in the spleen) generated from remaining progenitor cells from the embryonic life [31,36].

Interestingly, MDSCs in diverse sites differ from each other. MDSCs in the spleen produce higher amounts of reactive oxygen species (ROS) and smaller amounts of nitric oxide (NO) and express less arginase 1 (Arg-1), while MDSCs in the TME show the opposite trend. These differences are attributed, according to Corzo et al., to several factors present in the TME including hypoxia via the hypoxia-inducible factor 1α (HIF-1α) [39]. The relation of MDSCs and TME is bidirectional. On the one hand, tumour cells express and release factors that induce the generation of MDSCs in the BM and their recruitment and accumulation at the tumour site, such as chemokine (C-C motif) ligand 2 (CCL2), CCL5, CXC chemokines, STATs, interleukin 10 (IL-10), transforming growth factor β (TGF-β), and vascular endothelial growth factor (VEGF), while on the other hand MDSCs play a crucial role in the formation of the pre-metastatic niche through secretion of soluble factors and exosomes resulting this way in a positive loop for their development [40,41,42,43,44].

High numbers of MDSCs can also be found in the placenta and the umbilical cord. These two sites have distinct types of MDSCs; the umbilical cord MDSCs display a more immature phenotype indicating their foetal origin, while the placental MDSCs are of maternal origin. Umbilical cord MDSCs, and mainly the PMN-MDSCs, are generated from the abundant progenitor cells of the umbilical cord blood and are normally expanded to sustain the essential foetal–maternal tolerance [18]. According to Zhang et al., the human trophoblast cells can induce the development of a novel CD14^+^HLA-DR^-/low^ MDSC population from peripheral CD14^+^ myelomonocytic cells [45].

However, as far as the M-MDSCs are concerned, the expression of CD14, CD80, and CD83, their mature-like morphology, and the fact that they can differentiate into tumour-associated monocytes (TAMs) and dendritic cells (DCs), raises questions on the theory described above and it is believed by some experts that they are in fact re-programmed mature monocytes, under the signals of hypoxia or damage-associated molecular patterns (DAMPs) and pathogen-associated molecular patterns (PAMPs) [31,46,47]. At this point, it would be useful to note that M-MDSCs differ from TAMs. TAMs are mature macrophages of the TME and are divided in two categories, the M1 that have anti-tumoural activities and the M2 that have pro-tumoural activities. Large numbers of M-MDSCs are correlated with more advanced neoplastic disease, worse outcome and prognosis, and poor response to treatment, while large numbers of M2 predispose for poor outcomes and prognosis in patients with cancer but this cannot be stated for TAMs in general. TAMs share common markers and mechanisms of action with M-MDSCs but also have differences. Both cell populations express Arg-1, inducible nitric oxide synthase (iNOS), programmed death-ligand 1 (PD-L1), CD80, CD86, CD11b, C-C chemokine receptor type 2 (CCR2), and TGF-β, and secrete immune-suppressive cytokines such as IL-10, while TAMs express more intensely CD115 and interferon regulatory factor 8 (IRF8), but not S100A9 [24,38,48,49,50,51].

A lot of debate has also been over the actual presence of PMN-MDSCs. Some researchers still think that these cells are not a distinct type of cell, but rather an activated form of neutrophils with an immunosuppressive character and a de-granulated form and, resultantly, low-density [52], while others that they derive from re-programmed mature neutrophils [53,54]. Moreover, it is mentioned in the literature that M-MDSCs under the appropriate conditions can differentiate into PMN-MDSCs [55]. Similarly as with TAMs and M-MDSCs, it needs to be stressed that PMN-MDSCs are a different cell population from tumour-associated neutrophils (TANs). TANs are thought to exist, also, in two states, i.e., the N1 anti-tumoural and the N2 pro-tumoural subpopulations, but their distinction from PMN-MDSCs is still challenging as they share the same phenotypical markers, and further research is needed to make it possible [24,56].

**Table 1:** Examples of studies investigating the origin of MDSCs.

Study	Model	Theory	Generated Cells
Park et al. [32]	Human UCB cells cultured with rh-GM-CSF/SCF	MDSCs derived from UCB progenitor cells	Compatible with M-MDSCs
Pena et al. [57]	Endotoxin sepsis model; LPS-treated PBMCs	Re-programming of mature monocytes	Compatible with M-MDSCs
Singel et al. [58]	Human ovarian cancer cells	Re-programming of mature neutrophils	Compatible with PMN-MDSCs
Sinha et al. [59]	Tumour-bearing mice; PGE2-treated BM cells	BM haematopoiesis	Compatible with MDSCs
Song et al. [60]	Tumour-bearing mice	Extramedullary haematopoiesis	Compatible with MDSCs
Wynn [55]	Tumour-bearing mice	M-MDCs transformation to PMN-MDSCs	Compatible with PMN-MDSCs
Yu et al. [61]	Human UCB cells co-cultured with human breast cancer cells	MDSCs derived from UCB progenitor cells	Compatible with eMDSCs

Abbreviations: MDSCs: myeloid-derived suppressor cells; UCB: umbilical cord blood; rh-GM-CSF/SCF: recombinant human–granulocyte macrophage-colony stimulating factor/stem cell factor; LPS: lipopolysaccharide; PBMCs: peripheral blood mononuclear cells; PGE2: prostaglandin E2; BM: bone marrow.

## 4. What Are the Mechanisms of Action and the Molecular Pathways Correlated to MDSCs?

**Figure 2:**
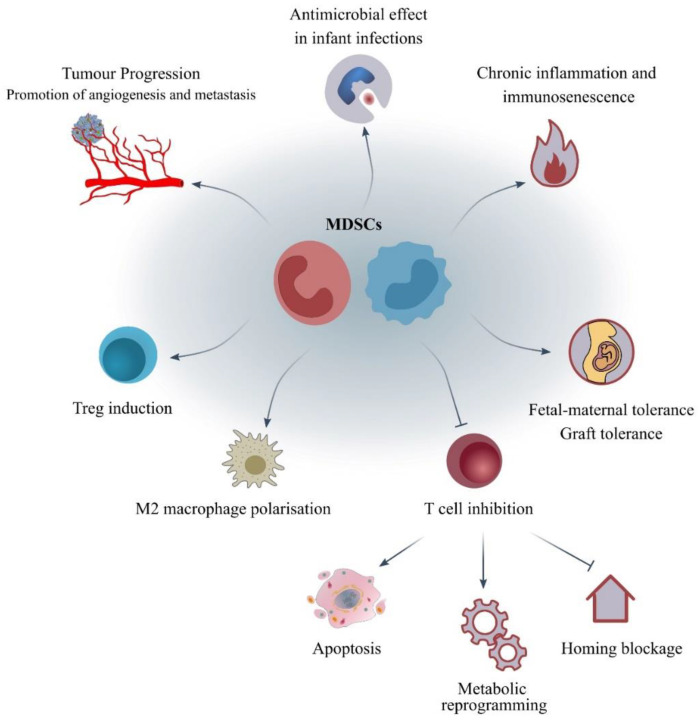
Mechanisms of action of MDSCs. MDSCs are thought to be cells with immune-modulatory properties. These cells are thought to be elevated and play an important role in chronic inflammation, tumour progression, and immunosenescence. They act through several mechanisms such as inhibition of T-cell responses, induction of T-regs, M2 macrophage polarisation, etc. However, they have also beneficial roles such as in foetal–maternal tolerance and during infant infections. Abbreviations: MDSCs—myeloid-derived suppressor cells; T-regs—T regulatory cells.

Recent literature tends to characterise MDSCs more as immune-modulatory rather than immune-suppressive cells, as their exact role and functionality seems to be driven by the microenvironment and the specific conditions under which they expand. Below, we summarise the main mechanisms of their actions (Table 2 and Figure 2). However, a more detailed review of the current literature concerning the mechanisms of immunosuppression mediated by MDSCs is provided by Vanhaver et al. in this Special Issue [62].

In neoplastic diseases, MDSCs are thought to be an important part of the so-called pre-metastatic niche that drives metastases of the primary lesion to remote organs. Among other factors, the expression of MMP9, CCL2, S100A8/9, VEGF, and BV8 facilitate new-angiogenesis and metastasis [63]. Interestingly, novel studies demonstrate that exosomes derived from MDSCs that carry proteins, miRNAs, and mRNAs are key players in the tumour development and evasion [63,64,65].

As important immunosuppression mediators, MDSCs promote their actions by either facilitating or hindering the functions of other immune cell populations. In some cases, they boost the activity of other immunosuppressive cells. For example, they induce the development of FoxP3^+^ T-regs through the production of INF-γ, IL-10, and TGF-β, as well as the M2 polarisation of macrophages [66].

In other cases, they demonstrate deleterious effects on other immune cells. Interestingly, MDSCs create a microenvironment of cytotoxic oxidative stress that leads to T-cell apoptosis. They express NOS, with PMN-MDSCs acting via endothelial NOS (eNOS) that increases peroxynitrites and M-MDSCs via iNOS that increases NO [5,67]. They also lead to the production of ROS, via NADPH oxidase isoforms (NOX), that act directly on other immune cells as well as indirectly through augmenting the further induction and recruitment of MDSCs [66].

Among others, MDSCs play an important role in the so-called energy metabolic reprogramming. Metabolomic analysis is also useful in the identification of MDSCs, as they demonstrate a different profile of metabolites in primary pathways from other immune cells, such as monocytes, neutrophils, DCs, and TAMs, while differentiations in metabolic pathways can lead to the maturation and differentiation of MDSCs to those cell types [13]. Energy metabolism affects MDSCs and therefore the TME. Under conditions of normoxia, MDSCs prefer lipolysis and fatty acid oxidation as well as oxidative phosphorylation, while under hypoxia via the upregulation of HIF-1α, glycolysis is increased. Glycolysis is also related to the maturation of MDSCs to TAMs and DCs [14,68]. It is important to note that mature monocytes and neutrophils are highly dependent on anaerobic glycolysis and that HIF-1α is therefore important for their activity. The switch from oxidative to glycolytic metabolism is associated with the activation of these cells [69,70,71].

Amino-acid (AA) metabolism plays an essential role in the functionality of MDSCs, especially the suppression of T-cells, as they express molecules that catabolise essential to T-cells AA. MDSCs reduce the levels of arginine (Arg) by expressing Arg-1 and iNOS, the levels of tryptophane (Trp) by expressing indoleamine 2,3-dioxygenase 1 (IDO1), and the levels of cysteine (Cys) by expressing an importer membrane protein but not an exporter [14,68]. Without Arg, T-cells cannot function as CD3ζ is nitrosylated and downregulated. The deprivation of essential AAs for T-cells leads to the blockage of their proliferation and survival [5,72,73]. Expression of ectoenzymes also leads to production of adenosine that inhibits T-cells [66].

Lang et al. were the first to compare the three major subpopulations of MDSCs with regards to their frequency, T-cell suppressive properties, and clinical significance in human cancer, and showed the superiority of PMN-MDSCs, compared to M-MDSCs and eMDSCs, for T-cell suppression and correlation with survival, while they further highlighted the CD11b^+^CD16^+^ subset of the PMN-MDSC subpopulation as demonstrating the strongest suppression capacity and Arg-1 expression [74]. Cassetta et al. further demonstrated the predominant expansion of PMN-MDSCs in solid tumours compared to infectious and inflammatory diseases [75].

MDSCs further sabotage the actions of T-cells by blocking their homing, as they express ADAM17, a metalloprotease that cleaves L-selectin (CD62L) [66]. Moreover, MDSCs express negative immune checkpoint molecules and via these molecules T-cells are suppressed in a contact-dependent manner. Ballbach et al. as well as other study groups have demonstrated the importance of PD-L1, expressed by MDSCs, and programmed death 1 receptor (PD-1), expressed by T-cells, with their interaction leading to the apoptosis of the effector cells [73,76].

Autophagy, when dysfunctional, displays an important pathway for the tumour progression. During this complex process, the mechanistic target of rapamycin complex 1 (mTORC1) and the AMP-activated protein kinase (AMPK) facilitates the initiation step, while the nucleation step leads to the activation of the Beclin-1–VPS34 complex. These two steps form the autophagic vesicle membrane followed by the maturation step with the autophagosome formation and the autophagic cargo uptake and the degradation step with the fusion with lysosomes and the exposure of the cargo to hydrolases [77,78]. Alissafi et al., in their novel study with samples from melanoma patients and mouse models, have shown the implication of autophagy in the suppressive character of M-MDSCs and in their immaturity associated with the decreased expression of surface major histocompatibility complex (MHC) II [8]. Dong et al. have also described the importance of autophagy in the accumulation and functionality of PMN-MDSCs, but their results are not in agreement with the previous group. The authors inhibited the pathway of autophagy in a mouse model and showed that this led to increased accumulation and suppressive function in vitro and in vivo via upregulation of STAT3 signalling and increased ROS production [79]. Further research is needed to clarify the role of autophagy in the biology of the subpopulations of MDSCs.

On the other hand, in certain cases, MDSCs also present beneficial roles, such as antimicrobial properties, a particularly important phenomenon in newborns [4]. Leiber et al. showed that neonatal PMN-MDSCs are capable of phagocytosing bacterial pathogens, while maintaining their immunosuppressive properties [80].

**Table 2:** Overview of the mechanisms of the immunosuppressive action of MDSCs.

Mechanisms of Action of MDSCs	Pathways/Molecules	Cells Affected/Impacts
Energy metabolic reprogramming	Arg-1 and iNOS: deprivation of Arg, IDO1: deprivation of Trp, Cys importer: deprivation of Cys	Deprivation of metabolites leading to T-cell suppression [14,68]
Autophagy	mTORC1, STAT3	Induction of the immaturity, suppressive character and accumulation of MDSCs [8,79]
Extracellular vesicle (EV) cargo	Exosome formation containing: miRNAs, mRNAs, dsDNA	Tumour development and evasion [64,65]
Induction of immunosuppressive cells	IFN-γ, IL-10, TGF-β	Induction of T-regs, polarisation of M2-like phenotype of macrophages [66]
Oxidative stress environment	iNOS: production of NO; eNOS: production of peroxynitrites; NOX: production of ROS	T-cell apoptosis [66,67]
T-cell homing blockage	ADAM17: a metalloprotease that cleaves L-selectin (CD62L)	Impairment of T-cell actions [66]
Angiogenesis and metastasis	MMP9, CCL2, S100A8/9, VEGF, BV8	Formation of the pre-metastatic niche [63]
Expression of negative immune checkpoint molecules	PD-L1/PD-1	T-cell suppression in a contact-dependent manner [73,76]
Expression of ectoenzymes	Adenosine	T-cell suppression [66]

Abbreviations: Arg-1: arginase-1; iNOS: inducible nitric oxide synthase; Trp: tryptophan; Cys: cysteine; mTORC1: mammalian target of rapamycin complex 1; STAT3: signal transducer and activator of transcription 3; IFN-γ: interferon γ; IL-10: interleukin 10; TGF-β: transforming growth factor β; T-regs: T regulatory cells; EV: extracellular vesicle; miRNA: microRNA; mRNA: messenger RNA; dsDNA: double-stranded DNA; eNOS: endothelial NOS; NOX: NADPH oxidase isoforms; ROS: reactive oxygen species; ADAM17: A disintegrin and metalloprotease 17; CD: cluster of differentiation; MMP9: matrix metallopeptidase 9; CCL2: chemokine (C-C motif) ligand 2; S100A8/9: S100 calcium-binding protein A8/9; VEGF: vascular endothelial growth factor; PD-L1/PD-1: programmed death-ligand 1/programmed death 1 receptor.

## 5. How Is the Phenotypic and Functional Analysis of Human MDSCs Performed?

### 5.1. Phenotypic Analysis

An important subject of discussion is the methods used to identify and analyse these cells. Unfortunately, there are still several obstacles, especially for clinicians, to proceed with routine measurement of MDSC populations in their patients. Despite the abundance of studies and meetings aiming to establish a universally accepted methodology, a high divergence between different laboratories is still present. In general, difficulties focus on defining cell-surface markers and gating strategies that uniquely identify the different populations of human MDSCs. This is especially problematic in myeloid malignancies, where increased immature myeloid cells are present [24,81,82].

In addition, many factors have to be taken into account, such as the time between the collection of the blood and the measurement of the cells (ideally within one hour but it can be extended up to 24 h in some studies), the temperature under which the samples are kept (room temperature versus 4 °C), the anticoagulant (ideally ethylenediaminetetraacetic acid (EDTA) or sodium citrate are recommended), and the separation reagent (gradient solution 1.077 g/L is recommended). Additionally, whether or not a dead-cell marker is used to exclude the dead cells is not well established in the literature. Probably, in cryopreserved or thawed samples or samples that have waited for more than the ideal time before the measurement of the cells, a dead-cell marker could be useful. However, this type of handling is not recommended [81,82,83,84]. Mandruzzato et al., in their proficiency panel study with 23 participating laboratories, found that the use of a dead-cell marker significantly altered the inter-laboratory but not the intra-laboratory variance [81]. Moreover, Cassetta et al. published the study Mye-EUNITER MDSC Monitoring Initiative (Mye-MMI) from 13 centres in an attempt to harmonise the protocols of the isolation and analysis of MDSCs between different laboratories [82].

The gold-standard method is thought to be the measurement of MDSCs in the fraction of PBMCs, in order to distinguish PMN-MDSCs, which are low-density neutrophils, from their mature normal density counterparts [24,85]. Interestingly, lectin-type oxidised LDL receptor-1 (LOX-1) has been recently proposed by Condamine et al. as a novel marker to distinguish PMN-MDSCs from mature neutrophils even from whole blood [86].

Overlapping immunophenotyping and functional features make the differentiation between PMN-MDSCs and tumour-associated neutrophils (TANs) and between M-MDSCs and tumour-associated macrophages (TAMs) challenging [87]. Although mature monocytes, which are divided into classical, intermediate, and non-classical subtypes, and are implicated in various pathological conditions, are also found in the fraction of PBMCs, they are easily differentiated from M-MDSCs using the HLA-DR expression. Interestingly, M-MDSCs tend to express CD33 more intensely compared to PMN-MDSCs, but it has to be stressed that CD33-negative neutrophils are not PMN-MDSCs and show no suppressive character [24].

The main problem seems to be the absence of a single marker for MDSC identification and distinction from other cell types, while the time-worthy method makes it difficult to use them as potential biomarkers in everyday clinical practice. Apodaca et al. have recently standardised a gating strategy with antibodies against 11 markers (i.e., CD45, CD3, CD19, CD20, CD56, CD16, HLA-DR, CD33, CD11b, CD14, and CD15) that they claim can lead to reliable results [88].

It is worth mentioning that Zoso et al. and Mazza et al. have generated the so-called fibrocytic MDSCs (f-MDSCs). These cells express the same time markers of MDSC, tolerogenic DCs (tDCs), and fibrocytes, i.e., CD33, IL-4Rα, CD11b, CD11c, CD13, CD14, CD15, HLA-DR, CD86, CD40, collagen V, and a-smooth muscle actin (a-SMA) [33,89].

As MDSCs originate from the differentiation of myeloid cells on the background of inflammatory, neoplastic, or autoimmune pathological states, they share common cell-surface markers with other cells of myeloid lineage, such as monocytes, macrophages, and granulocytes. Thus, for a cell to be characterised as an MDSC, apart from its immunophenotypic characterisation by identifying a specific constellation of cell-surface markers, its T-cell suppressive properties need to be additionally demonstrated by means of functional assays [25]. MDSC-induced suppression of T-cells is mediated by multiple immunosuppressive mechanisms, and the implicated molecular factors could potentially be used as surrogate markers whose analysis could be performed in parallel with the standardised functional assays in challenging samples [75,82].

In Table 3 we aim to highlight the main important markers and molecules that can facilitate the clinician and researcher to identify and study MDSCs.

### 5.2. Functional Analysis

Functional assays are interwoven with the study of MDSCs as, alongside their immunophenotype, their suppressive character must be further proven. However, these assays are challenging, especially for human MDSCs, because of the absence of a single marker showing activity of these cells, the sensitivity and small numbers of the cells, and the inability to test in vivo their activity in human individuals. Several different methods are used, which involve the direct detection via flow cytometry of markers indicating functionality (e.g., LOX-1), the detection of products of the MDSCs pathways (e.g., NO, ROS, IL-10, and TGFβ), the differentiation in the levels of molecules that MDSCs cause the deprivation of (e.g., Arg, Cys), the upregulation of molecules involved in these pathways (e.g., Arg1, IDO, iNOS, PD-L1, CD39, CD73, and Fas ligand) and transcription factors (e.g., STATs), and the study of the effect of MDSCs on the T-cell functionality (e.g., proliferation, IFN-γ production, FoXP3 expression, CD3ζ chain expression, nitration of TCR, and IL-2 production) [25,77].

Although MDSCs affect a lot of different cell types involving NK, macrophages, and T-regs, the T-cell suppression assay is the most commonly used technique. However, there are many differences in the protocols of each laboratory. Firstly, some laboratories use a system that is autologous, i.e., T-cells and MDSCs from the same individual, while others one that is allogenic, i.e., T-cells and MDSCs from two different individuals [25]. Secondly, cell proliferation can also be measured in several different ways, which include carboxyfluorescein succinimidyl ester (CFSE) staining, which is mostly used, and Ki-67 expression and 3H-thymidine incorporation, which are rarely used because of financial issues and issues of reliability and sensitivity, respectively [25,111]. Thirdly, another step that differs between laboratories is the stimulants of proliferation, which could be IL-2, phytohaemagglutinin (PHA), or anti-CD3/anti-CD28 stimulation [25]. Solito et al. described nine basic protocols of measuring the immune suppressive activity of MDSCs both in vitro and in vivo, while Bruger et al. highlighted the main methods and the accompanying difficulties [112].

This aforementioned diversity in the protocols of T-cell-suppression assays and thus the different ways of interpreting the results, led to the need for a harmonised protocol. The COST initiative Mye-EUNITER ran a project called Mye-FUN in order to meet this need. To harmonise the T-cell-suppression assays used for studying the functionality of MDSCs, Bruger et al. published in 2019 their protocol that was the result of this initiative. Their protocol involves allogeneic MDSCs and T-cells. The MDSCs are isolated through flow cytometry cell sorting, while the T-cells used come from donors without neoplastic disease and are frozen in order to be used between different laboratories and in different timepoints. Autologous-to-T-cells DCs are added to facilitate their proliferation. As stimulants of cell proliferation, the authors used anti-CD3/anti-CD28 antibodies. The dye used to track the cell proliferation is not predefined by the authors, and CFSE and CMFDA are provided as examples [113].

## 6. Which Are the Key Points of Studying MDSCs in Haematology?

### 6.1. Increased MDSCs: Haematologic Malignancies and Myelodysplasia

As reviewed earlier [28], MDSCs have been studied in many haematologic diseases. In malignancies and dysplasia, MDSCs are generally found at higher levels and related to the abnormal BM niche. More specifically, there are studies on increased PMN-MDSCs, mostly in patients with myeloproliferative neoplasms (MPNs), i.e., chronic myeloid leukaemia (CML), polycythemia vera (PV), essential thrombocythemia (ET), and primary myelofibrosis (PMF). In all MPNs, MDSCs are found to express Arg-1 in higher amounts [114]. Especially in CML, these cells contribute to the immune escape and, according to studies, there is a bidirectional relation with the leukemic cells, as cells from patients can convert monocytes from healthy donors into cells with the MDSC-suppressive character [115]. In rather limited studies, PMN-MDSCs seem to accumulate in acute myeloid leukaemia (AML) and acute lymphoblastic leukaemia (ALL) [28,116,117], while M-MDSCs seem to accumulate in chronic lymphocytic leukaemia (CLL) [118,119,120,121,122]. In AML, the mucin 1 (MUC1) oncoprotein is shown to facilitate the proliferation of MDSCs [123], and there seems to be a correlation between increased numbers of this population and extramedullary involvement, plasma D-dimers, higher minimal residual disease (MRD), blast cell frequency, and Wilms 1 (WT-1) gene detection [124]. In myelodysplastic syndromes (MDS), more research is needed, but several studies showed increased MDSC populations, upregulated related pathways and molecules, and a positive correlation with the T-regs population [125,126,127]. Moreover, in MDS, the expression of S100A8/A9 and its interaction with CD33 seems to play an important role in the induction of dysplasia [127]. The MDSC populations were also expanded in a number of studies for multiple myeloma (MM) [28], where MDSCs seem to attenuate the survival of MM cells in several ways, such as via the phosphorylation of 5’ adenosine monophosphate-activated protein kinase (AMPK) [128], with PMN-MDSCs being the main population [129]. Ramachandran et al. showed that both BM PMN-MDSCs and neutrophils from mice with MM attenuated via soluble factors the survival of MM cells treated with chemotherapy [130], while according to Binsfeld et al., PMN-MDSCs present an upregulation in pro-angiogenic factors in the context of MM [131]. Lymphomas, including both Hodgkin lymphomas (HL) and non-Hodgkin lymphomas (NHL), are also correlated with increased numbers of M-MDSCs and PMN-MDSCs [132,133,134,135]. Especially in diffuse large B-cell lymphoma (DLBCL), M-MDSCs showed a positive correlation with T-regs [136].

### 6.2. Decreased MDSCs: Immune-Mediated Cytopenias

A decreased number of MDSCs is related with fewer conditions, such as immune-cytopenias. In our on-going study in the Haemopoiesis Research Laboratory at the University of Crete, we have found this to be true for patients with chronic idiopathic neutropenia (CIN) (also known as idiopathic neutropenia of undetermined significance, ICUS-N) [137], which correlates with the immune dysregulation accompanying the disease [138]. The decreased numbers of MDSCs are likely in part responsible for the chronic inflammation in these patients. Although, as pointed out by Bizymi et al., the frequency of MDSCs in immune thrombocytopenia (ITP) is still controversial [28], their contribution in the pathophysiology of the disease is undoubted [139]. In the small numbers of studies, MDSCs seem to decrease and have reduced immunosuppressive properties, such as downregulation of Arg-1, with disease activity [140]. However, Shao et al. showed that newly diagnosed patients present higher numbers compared to healthy controls [141,142], although those expanded cells in the start of the pathology may not have immunosuppressive properties as Weng et al. hypothesised [143].

### 6.3. MDSCs versus Mesenchymal Stem Cells (MSCs)

Both MDSCs and MSCs are immature cell populations with immunomodulatory properties (through common and different mechanisms) and are activated by the same factors, although they originate from distinct differentiation lines. They block the maturation of DCs and macrophages, antigen presentation, T-cell proliferation, Th1 responses, and NK cell action via common molecules, such as IDO, prostaglandin E2 (PGE2), IL-10, and TGF-β. However, they possess many differences as well. Only MDSCs produce Arg-1 and ROS, while the production of galectins and HLA-G is a unique feature of MSCs. MDSCs seem to be more sensitive to type 2 cytokines. MSCs foster the development and survival of neutrophils, in contrast to MDSCs that seem to react negatively to the neutrophilic differentiation. However, there is still limited data on the actual effect of MDSCs on neutrophils and more study is needed [144]. More detailed analysis of the interactions of MDSCs and MSCs in myeloid malignancies is provided by Kapor et al., in the context of this Special Issue [145]. 

### 6.4. Umbilical Cord Blood Subsets

Since allografts transplanted in haematologic patients can be from cord blood stem cells, the nature of MDSCs in cord blood is of great importance. MDSCs are supposed to play an important role for the foetal–maternal tolerance and decreased numbers of them are associated with miscarriage [146,147]. In agreement to this hypothesis, several studies proved the suppressive function of increased cord blood PMN-MDSCs. The number of M-MDSCs was unchanged [148,149,150]. Kostlin et al. also found PMN-MDSCs accumulated in the healthy placenta and polarised T-cells to Th2 responses [17]. Moreover, a recent study conducted by Schwarz et al. showed increased levels of PMN-MDSCs in the cord blood of preterm infants. The increase was sustained in the peripheral blood during the neonatal period [151]. Zoso et al. and Mazza et al., as aforementioned, expanded ex vivo the distinct novel subpopulation of MDSCs that originated from umbilical cord blood precursors, named f-MDSCs. The authors propose this novel subset as a tool for treatment of allograft rejection and in vitro generation of T-regs [33,84]. A detailed review of the literature is provided by Bizymi et al. in the context of this Special Issue [152].

## 7. What Are the Potential Clinical Applications of MDSCs in Haematology?

### 7.1. MDSCs as Biomarkers

MDSCs are found to be increased in a number of studies in patients suffering from MDS, MPN, MM, lymphomas, and leukaemia. This increase is associated in most cases with poor outcome, relapsed and refractory disease, and higher risk of therapy failure. Thus, the levels of MDSCs may work as biomarkers of diagnosis, disease activity, response to treatment, or disease progression, and there are a number of studies in this direction in the literature [28].

All subtypes of MDSCs seem to have increased numbers in HL, as well as in NHL and MM. However, the studies concerning MDSCs in NHL and MM are diverse and still share contrary results. Interestingly, in all studies, the patients with higher numbers of these populations also have poorer markers of survival and worse disease progression [134]. For example, Wang et al. described in their original study that elevated levels of circulating M-MDSCs are correlated to tumour progression and poorer overall survival in patients with DLBCL [153]. Additionally, Wu et al. described the prognostic value of M-MDSCs in DLBCL [154]. A special mention of MDSCs and lymphoid malignancies is provided in the context of this Special Issue as Papafragkos et al. have published their detailed review concerning this subject [155].

In the study of Kittang et al., MDSCs seemed to be higher in high-risk patients compared to lower risk, indicating that these cells may serve as biomarkers of severity in myelodysplasia in the future [125]. In the novel study by Geskin et al. including patients with mycosis fungoides (MF) and Sézary syndrome (SzS), MDSC activity, as evaluated by the ROS production, increased with the activity of the disease, i.e., it was higher in patients with >IB MF than in IA stage, although the numbers of MDSCs did not differ among the study groups [156]. However, more research on the field is needed in order to establish whether MDSCs are related to severity in T-cell malignancies.

Despite the small number of studies and the contradictory results in ITP as explained above, all studies agree that an increase in MDSCs is a common finding in remission, indicating that MDSCs can be a biomarker of disease activity and therapeutic response [140,141,142].

### 7.2. MDSCs as Therapeutic Targets

MDSCs are promising therapeutic targets. Up to now, the strategies have been focusing on depletion, deactivation, differentiation, or blockage of their development. The targeting of MDSCs functions synergistically with immunotherapy, leading to better results for the patient. Older as well as novel agents (all-trans retinoic acid (ATRA), IL-4, celecoxib, gemcitabine, etc.) seem to affect the number or properties of these cells, as they act on pathways essential to them as well [157,158,159,160,161,162,163,164]. Olivares-Hernández et al. reviewed in the context of this Special Issue the current literature on an interesting subject, i.e., how targeting MDSCs in haematologic malignancies can facilitate the therapy with immune checkpoint inhibitors (ICIs), as resistance to ICIs could be secondary to MDSCs [165].

Treatment with high-dose dexamethasone (DXM) restored the levels of MDSCs in patients with ITP in a dose-dependent manner [140,141,142,166]. In the experiments of Hou et al., MDSCs from ITP patients treated with DXM improved their capacity to suppress T-cells and upregulated the expression of Ets1. The authors transferred MDSCs to a mouse model of ITP and observed an increase in the platelet counts and Ets1 expression [139,166]. Similarly, intravenous immunoglobulin (IVIG) treatment improved the numbers of MDSCs in spleen cells of ITP patients and trauma patients [140,167].

Eksioglou et al. targeted MDSCs in low-risk MDS patients with an antibody against CD33. Blocking CD33 led to direct cell toxicity and cell death, as well as disruption of the downstream signalling and the interaction with S100A8/A9 [168]. In the aforementioned study of Geskin et al., the authors claimed that the treatment with IFN-a2b used in patients with MF owes in part its effect to preventing the immunosuppressive properties of MDSCs, as the treated individuals, although not changing their cell population numbers, presented decreased serum arginase levels and MDSC ROS production [156].

### 7.3. Graft-versus-Host Disease

An important step that historically boosted the research on MDSCs was their involvement in allogeneic haematopoietic stem cell transplantation (AHSCT), a crucial subject in haematology. It seems that they can enhance the amelioration of the graft-versus-host disease (GvHD), the state where the transplanted cells reject and attack the host. The observation that has led to this hypothesis was that the mobilisation of stem cells with G-CSF was correlated with an increase in MDSCs as well. Since then, several targeting strategies have been suggested and are under investigation, in order to eliminate the risk of GvHD in patients treated with AHSCT [169,170,171,172]. A paper by Demosthenous et al. was published in the context of this Special Issue dedicated to MDSCs in GvHD [173].

## 8. Synopsis and Future Perspectives

Despite the difficulties in defining cell-surface markers and gating strategies that uniquely identify the different populations of human MDSCs, there is a large list of haematologic conditions related to abnormal number and/or function of MDSCs. Research has revealed the strong association between increased populations of MDSCs and malignancies and states of chronic inflammation, such as autoimmunity and infections. Another condition where MDSCs are, in contrast, beneficial is the amelioration of GvHD in AHSCT, as here their suppressive phenotype can reduce the risk of the graft attacking the host.

However, there are still many controversial issues that need further investigation to be clarified. The signalling pathways involved have not been studied in detail, and the way that drugs affect MDSCs is unclear in most cases. Especially, eMDSCs are poorly studied and their impact is vague. Although the difficulties mentioned do exist, more targeted research strategies are needed to reveal a translational (from the bench to the bedside) outcome. In the future, ongoing research can lead to the development of therapeutic strategies targeting MDSCs as well as diagnostic strategies using them as biomarkers for follow-up of disease progression, estimation of response to treatment, and prediction of disease outcome.

In conclusion, the data obtained by studies of MDSCs in haematologic diseases show their importance in the pathogenesis of these conditions and thus the alteration of these cell populations. However, the study of MDSCs is not yet a routine practice in laboratories of clinical and experimental haematology, and the methods and protocols used are diverse and do not always reflect the official recommendations. Their broader introduction in the studies of such laboratories necessitates a better understanding of their biology and functionality and will facilitate their potential use as biomarkers of disease severity, response to treatment, and prognosis as well as therapeutic tools in the future. This review and this Special Issue intend to help researchers and clinicians in the field of haematology, interested in starting novel protocols concerning MDSCs, to familiarise themselves with the current literature and applicable methods in a precise, thorough, and concise manner, in order to further promote translational research on the different roles and applications of MDSCs in health and disease.

## Figures and Tables

**Table 3:** Markers and molecules important for the study and identification of MDSCs.

Marker/Molecule	Cell	Function	Importance in MDSCs
HLA-DR: Major histocompatibility complex (MHC) II cell surface receptor	Antigen-presenting cells	Antigen presentation to T-cells	Absent or low in MDSCs [24,90]
CD33	Expression decreases in mature cells, expressed in myeloid stem cells (CFU-GEMM, CFU-GM, CFU-G, E-BFU), myeloblasts, monoblasts, monocytes/macrophages, granulocyte precursors, and mast cells	Sialoadhesin	Present [24,91,92]
CD66b or CEACAM8 or CGM6 or NCA-95	Granulocytes, eosinophils (granulocyte lineage cells)	Regulation of adhesion and activation	Present in PMN-MDSCs [24,91,93,94]
CD11b	Granulocytes, monocytes/macrophages, NK cells, and subsets of B- and T-cells, pan-myeloid marker	Adhesion	Present [86]
CD11c	Monocytes/macrophages, NK cells, and hairy cells	Adhesion	Mostly absent [86,90]
CD45 (lymphocyte common antigen)	Leukocytes	Receptor-linked protein tyrosine phosphatase, activation, signal transduction	Present [42,91]
CD15 (Lewis x or Le^x^)	Present in cells past the myeloblast stage in the granulocytic lineage	Neutrophil adhesion to dendritic cells	Present in PMN-MDSCs [24,86]
CD14	Expressed in the myelomonocyte lineage (monocytes, macrophages)	Endotoxin receptor, activation of innate immunity	Present in M-MDSCs [24,92]
CD3	Mature T-cells	Part of the TCR complex	Absent [86]
CD19	B-cells, precursors of B-cells, follicular dendritic cells	Regulation of development, activation, and differentiation	Absent [86]
CD20	B-cells	Development and differentiation of B-cells into plasma cells	Absent [86]
CD16	Subsets of NK and T-cell types, granulocytes, tissue macrophages, CD16^+^ subsets of monocytes, eosinophils, DCs	Fc gamma receptor; antibody-dependent cell-mediated cytotoxicity	Absent [86]
CD56	Neural cells, NK cells, subset of T-cells	Adhesion molecule	Absent [86]
CD13	Mature neutrophils	Aminopeptidase N; mediates tumour angiogenesis	Present in CD13^high^ PMN-MDSCs [86,93,94]
CD40	Antigen-presenting cells, epithelial cells, hematopoietic progenitor cells, activated T-cells	Member of the tumour necrosis factor (TNF) receptor superfamily; regulates tumour growth and apoptosis dependently on the level of its expression; promotes the immunosuppression of MDSCs and T-regs induction but not their accumulation	Present in MDSCs [86,95]
CD80	Myeloid cells	Protein B7-1; suppression of T-cell immune responses; binds both CD28 (stimulatory) and CTLA-4 (inhibitory) on T-cells	Present in MDSCs [2,96]
CD86	Antigen-presenting cells	Protein B7-2; activation of T-cells; binds both CD28 (stimulatory) and CTLA-4 (inhibitory) on T-cells	Absent or low in MDSCs [96]
CD83	Antigen-presenting cells, mature DCs	Immune responses	Present in M-MDSCs [31,90]
CD36	Widespread presence	Fatty acid translocase; immunosuppressive activity of MDSCs	Present in MDSCs [14]
HLA-ABC: Major histocompatibility complex (MHC) I antigens	Widespread presence	Immune response	Present in MDSCs [2]
CD54 or ICAM-1 (Intercellular Adhesion Molecule 1)	Endothelial cells, leukocytes	Cell–cell interactions, transmigration	High in M-MDSCs, low in PMN-MDSCs [90]
CD195 or CCR5	Leukocytes	C-C chemokine receptor type 5	Present [86,90]
CD197 or CCR7	Mature DCs, B-cells, T-cells, cancer cells	C-C chemokine receptor type 7	Present [86,97]
CD62L or L-selectin	Leukocytes	Cell adhesion molecule	Present [2,86]
E-Cadherin	Epithelial cells, cancer cells	Cell adhesion molecule	Downregulated by MDSCs [98,99]
N-Cadherin	Mesenchymal cells, cancer cells	Cell adhesion molecule	Upregulated by MDSCs [99]
CD124 or IL-4Ra	B-cells, T-cells, NK cells, macrophages	Activation of STAT6, Th2 response	Present [2,26]
CD34 or Sialomucin	Early haemopoietic cells, endothelial cells	Cell adhesion molecule	Absent [2,86]
LOX-1	Endothelial cells, low expression in normal neutrophils	Receptor oxidised LDL receptor 1; endoplasmic reticulum (ER) stress, lipid metabolism	Present in PMN-MDSCs [81]
CD192 or CCR2	Leukocytes	C-C chemokine receptor type 2	High in M-MDSCs, low in PMN-MDSCs [90]
CXCR4	T-cells	Recruitment	Present [86,90]
Arg1	Erythrocytes, lymphocytes, macrophages	Deprivation of arginine that is crucial for TCR expression and TCR-mediated signal transduction	Present [100]
NO	Widespread presence	Nitration of TCRs and production of chemokines important for T-cell migration or induction of T-cell apoptosis	Present mainly in M-MDSCs [62]
ROS	Widespread presence	Oxidative stress induction, apoptosis of T-cells	Present [101]
S100A8/9	Myeloid cells, tumour cells	Interaction with CD33, NF-kB pathway induction, MDSCs migration	Present [102]
STATs	Immune cells, tumour cells	Transcription factors important for MDSCs development	Present [103,104]
PD-L1 or B7-H1 or CD274	Myeloid cells, lymphoid cells, epithelial cells, tumour cells	Co-inhibitory receptor leading to T-cell activity suppression	Present in M-MDSCs (mainly) and PMN-MDSCs [71]
C/EBPb	BM cells in differentiation of myeloid lineage and emergency granulopoiesis	Transcription factor important for immunosuppressive properties of MDSCs	Present [67,105]
TGF-β	Secreted by most cells	Inhibition of immune effector cell functions	Secretion by MDSCs [67]
IL-10	Secreted by immune cells	Inhibition of immune effector cell functions	Secretion by MDSCs [67]
VEGF	Secreted in TME	Angiogenesis	Induction of its secretion by MDSCs [67]
CD115	Monocyte and macrophage lineage cells	Receptor of M-CSF	Present [86]
CD244	NK cells, T-cells, monocytes, dendritic cells	Important for immunosuppressive properties of MDSCs	Present in PMN-MDSCs [106]
CD163	Activated T-cells, macrophages	Scavenger receptor	Present in M-MDSCs [41,107]
CD39	Leukocytes	Increased production of adenosine that suppresses effector T-cell function	Present [86,108]
CD73	B-cells, T-cells	Increased production of adenosine that suppresses effector T-cell function	Present [86,108]
Fas Ligand or CD95L or CD178	T-cells, NK cells	Mediator of T-cell apoptosis	Present in PMN-MDSCs [86,109]
IDO	Antigen-presenting cells, tumour cells	Degradation of L-tryptophan leading to cell cycle arrest and anergy of T-cells and differentiation of T-cells into T-regs	Present [110]

Abbreviations: MHC: major histocompatibility complex; CD: cluster of differentiation: CFU-GEMM: colony forming unit–granulocyte, erythrocyte, monocyte, megakaryocyte; CFU-GM: granulocyte–macrophage progenitor; E-BFU: erythroid–burst forming unit; CEACAM: CEA cell adhesion molecule 8; NK cell: natural killer cell; TCR: T-cell receptor; DC: dendritic cell; TNF: tumour necrosis factor; ICAM-1: intercellular adhesion molecule 1; CCR: C-C chemokine receptor; IL-4Ra: interleukin 4 receptor alpha; STAT: signal transducer and activator of transcription; LOX-1: lectin-type oxidised LDL receptor-1; ER: endoplasmic reticulum; CXCR: α-chemokine receptor; Arg1: arginase-1; NO: nitric oxide; ROS: reactive oxygen species; S100A8/9: S100 calcium-binding protein A8/9; PD-L1: programmed death-ligand 1; C/EBPb: CCAAT/enhancer-binding protein b; BM: bone marrow; TGF-β: transforming growth factor β; IL-10: interleukin 10; VEGF: vascular endothelial growth factor; TME: tumour microenvironment; M-CSF: macrophage colony-stimulating factor.

## Data Availability

Not applicable.
